# Data on nutritional awareness among Saudi adults: Al-Ahsa study

**DOI:** 10.6026/973206300223516

**Published:** 2026-06-30

**Authors:** BiBi Mariam, Suchithra Koipurathu Rajappan, Fatimah Ahmed AlMubark, Zahra'a Ahmed AlShayeb, Hawra'a Mohammed AlSalman, Zahra Sameer Al Abdi, Kholoud Hamdan Al Rawaili, Latifa Abdullah Al Jafaar

**Affiliations:** 1 Department of Clinical Nutrition, College of Applied Medical Sciences, King Saud Bin Abdulaziz University for Health Sciences, Al Ahsa 31982, Saudi Arabia; 2 Department of Basic Science, College of Science and Health Professions, King Saud Bin Abdulaziz University for Health Sciences, Al Ahsa 31982, Saudi Arabia; 3 King Abdullah International Medical Research Center, Al Ahsa 31982, Saudi Arabia; 4 Ministry of National Guard - Health Affairs, Al Ahsa 31982, Saudi Arabia

**Keywords:** Nutritional awareness, healthy lifestyle, non-communicable diseases (NCDs), dietary habits, gender differences, Saudi Arabia, Al-Ahsa

## Abstract

Nutritional awareness is essential for preventing non-communicable diseases (NCDs) linked to poor diet and sedentary lifestyles. In Saudi Arabia, gender-specific patterns of nutrition knowledge remain inconsistent, necessitating localized evidence to guide health promotion strategies. Therefore, it is of interest to assess and compare awareness of healthy nutritional practices among 538 Saudi adults aged 18-65 years in Al-Ahsa using a validated questionnaire. Overall nutritional awareness was moderate (males: 2.12 ± 0.16; females: 2.13 ± 0.17), while nutrition and physical-activity awareness were high in both genders. Mann-Whitney U tests showed no statistically significant gender differences in awareness scores (p > 0.05). Despite adequate awareness, unhealthy behaviors such as meal skipping (77.9%) and sedentary lifestyle (34.8%) were common. Thus, we show a gap between awareness and practice and support the need for targeted community nutrition interventions.

## Background:

Awareness of healthy nutritional practices is essential for the prevention of non-communicable diseases (NCDs) such as obesity, diabetes and cardiovascular diseases, which are strongly associated with unhealthy dietary patterns and sedentary lifestyles [1, 2, 3]. Globally, NCDs represent a major public health burden and contribute substantially to morbidity and mortality worldwide, with their prevalence continuing to rise due to rapid urbanization, dietary transitions and lifestyle changes [2, 3, 4, 5]. In Saudi Arabia, rapid socioeconomic development and the adoption of westernized dietary habits have contributed to a growing burden of obesity, diabetes and other metabolic disorders among adults, as supported by recent national studies [5, 6, 7, 8]. Previous studies in Saudi Arabia have reported moderate levels of nutrition knowledge and awareness among adults [9, 10, 11]. Additionally, a study conducted in Al-Ahsa reported moderate nutrition knowledge and highlighted the influence of socio-demographic factors on the use of nutrition information, emphasizing gaps between awareness and actual dietary practices [11]. Nutritional awareness plays a critical role in shaping dietary behaviors, including food selection, portion control and interpretation of nutritional information, which ultimately influence overall health outcomes. In response to the increasing burden of diet-related diseases, public health initiatives such as calorie labeling policies and national health campaigns have been implemented in Saudi Arabia to encourage healthier food choices and improve nutrition knowledge among the population. However, previous studies suggest that awareness alone does not necessarily translate into healthier dietary behaviors and a gap between knowledge and actual lifestyle practices is frequently observed, as ighlighted in recent literature [12, 13]. Furthermore, gender differences in nutritional awareness and dietary practices have been reported in several studies, with women often demonstrating greater engagement in health information and healthier dietary behaviors compared with men [11, 12, 13, 14]. Nevertheless, findings remain inconsistent across different populations and regions of Saudi Arabia [12, 13, 14]. Therefore, it is of interest to describe gender differences in nutritional awareness among Saudi adults in Al-Ahsa, Saudi Arabia.

## Methodology: 

A cross-sectional study was conducted in Al-Ahsa, Saudi Arabia, between July and November 2021 to assess gender differences in nutritional awareness among adults. Cross-sectional designs are widely used in public health research to evaluate knowledge, attitudes and health-related behaviors within a defined population at a specific point in time. Adults aged 18-65 years were recruited using random sampling techniques. A total of 538 participants were included in the study, exceeding the minimum required sample size of 384 calculated based on standard epidemiological sample size estimation methods for population-based surveys [3]. Data were collected using a validated and pretested questionnaire distributed through online platforms and virtual interviews. The questionnaire included sections addressing demographic characteristics, health status, dietary habits and awareness of nutrition and physical activity. The development and use of structured questionnaires for assessing dietary knowledge and nutrition awareness have been widely reported in previous public health nutrition studies [11]. Ethical approval for the study was obtained from the King Abdullah International Medical Research Center (KAIMRC: SP21A/371/07) and all participants provided informed consent prior to participation. The reliability of the questionnaire was evaluated using Cronbach's alpha to ensure internal consistency of the measurement tool. Data were analyzed using the Statistical Package for the Social Sciences (SPSS) version 22. Descriptive statistics were used to summarize participant characteristics and the Mann-Whitney U test was applied to compare gender differences in nutritional awareness scores. A p-value of <0.05 was considered statistically significant.

## Results:

A total of 538 respondents participated, including 32% males and 68% females. Most participants were aged between 18-45 years and 54.8% held graduate-level education. Approximately 41.3% were employed Table 1. Moderate physical activity was reported by 41.3% of participants, whereas 34.8% were classified as sedentary Table 2. The majority of participants were non-vegetarian (78.6%) and 77.9% reported skipping meals at least sometimes Table 3. Table 4 shows that the mean weight of males was 84.1 kg (SD = 21.2), whereas the mean weight of females was 65.4 kg (SD = 20.0). Both genders showed relatively large variability in body weight. The mean height was 171.8 cm for males and 157.5 cm for females, with standard deviations of approximately 7 cm. Figure 1 (see PDF) shows that mass media was the primary source of nutrition information for both genders. Female participants reported nutritionists and books as important additional sources of knowledge, whereas males relied more on friends and relatives. The overall level of awareness of healthy nutritional practices was moderate. The weighted mean score for males was 2.12 ± 0.16, while for females it was 2.13 ± 0.17. Awareness of nutrition and physical activity was high in both genders. The mean nutritional awareness scores were 2.64 ± 0.20 for males and 2.67 ± 0.20 for females. Similarly, physical-activity awareness scores were 2.36 ± 0.27 for males and 2.36 ± 0.24 for females. The results are presented in Table 5 and Figure 2 (see PDF). Whereas men seem to refer to friends and relatives to be their prime source of knowledge over nutritionists and books. The overall level of healthy nutritional awareness was moderate on healthy nutrition practices where the weighted mean for males was (2.12) with (0.16) standard deviation, where the female weighted mean was (2.13) with a standard deviation of (0.17). The level of healthy nutritional awareness was moderate and level of nutritional as well as physical awareness was high in the males and females, where the weighted means were (2.64) and (2.67) with same standard deviation of (0.20) for nutritional awareness for males and females respectively. Moreover, the weighted means were same (2.36) and with a standard deviation of (0.27) and (0.24) for physical activity awareness for males and females respectively. There is a significant difference in the awareness female and male results are presented in Table 5. The results of the Mann-Whitney U test Table 6 shows that there is no statistically significant difference between male and female in their level of mean awareness score in all three categories (p<0.05) (Healthy nutrition practices, Nutrition, Physical activity). The Mann-Whitney U value for healthy nutrition practices was (28341.5) with a significance level of (0.06), the value of nutrition was (27811) with a significance level of (0.28) and the value of physical activity was (30710) with a significance level of (0.647). Therefore, there are no statistically significant differences in healthy nutritional awareness between men and women in Al-Ahsa city. The overall awareness of healthy nutritional practices was moderate for both males (mean = 2.12 ± 0.16) and females (mean = 2.13 ± 0.17). Awareness of nutrition and physical activity was high in both genders. The Mann-Whitney U test showed no statistically significant differences between males and females in any awareness category (p > 0.05). Figure 2 (see PDF) shows that 59.78% of female participants reported having a chronic disease, whereas 40.22% of male participants reported chronic disease.

## Discussion:

The present study evaluated gender differences in nutritional awareness among Saudi adults in Al-Ahsa. The findings indicate that the overall awareness of healthy nutritional practices among the study population was moderate, while awareness related to nutrition and physical activity was relatively high among both males and females. The findings of the present study are consistent with previous research conducted in Al-Ahsa, which demonstrated moderate levels of nutrition knowledge and identified a gap between awareness and practice, despite reasonable awareness of nutrition-related information [11]. This supports the notion that knowledge alone may not lead to behavioral change, as also reported in recent studies highlighting a gap between nutrition knowledge and dietary practices [11, 12, 15]. These findings are consistent with previous studies conducted in Saudi Arabia that reported moderate levels of nutrition knowledge and dietary awareness among adult populations [9, 11, 14]. Despite relatively acceptable awareness levels, unhealthy behaviors such as meal skipping and sedentary lifestyles were commonly observed among participants. Similar observations have been reported in previous studies, which highlighted a gap between nutrition knowledge and actual dietary practices among adults. This discrepancy suggests that improving awareness alone may not necessarily translate into sustained behavioral change without supportive environmental and behavioral interventions [11, 12, 14]. This indicates that awareness alone is insufficient and underscores the need for behavior-focused and policy-driven interventions to promote sustainable lifestyle changes [12, 16]. Interestingly, the present study did not identify statistically significant gender differences in nutritional awareness. This finding contrasts with several earlier and recent studies reporting that women tend to demonstrate higher levels of nutrition knowledge and greater engagement in healthy dietary practices compared with men [10, 14]. However, recent research conducted among Saudi populations has also indicated minimal or no significant gender differences in nutritional awareness levels, suggesting that access to health information may now be more equally distributed between genders [14, 16]. Mass media and digital platforms were identified as the primary sources of nutrition information, consistent with recent studies highlighting the growing role of social media in shaping dietary behaviors. This observation is consistent with previous studies indicating that television, internet platforms and social media have become major channels for disseminating health information and influencing dietary behaviors among adults [13, 17]. Therefore, public health initiatives should continue to utilize digital communication platforms and community-based educational strategies to promote evidence-based nutrition awareness. Furthermore, the relatively high proportion of participants reporting chronic health conditions, particularly among female respondents, aligns with national reports documenting the growing burden of non-communicable diseases in Saudi Arabia. These findings align with recent national data indicating an increasing burden of non-communicable diseases in Saudi Arabia [7, 8]. These findings emphasize the importance of implementing comprehensive public health strategies aimed at improving dietary behaviors, promoting active lifestyles and strengthening nutrition education programs within the community [18].

## Conclusion:

Adults in Al-Ahsa exhibit moderate nutritional awareness but continue to engage in unhealthy behaviors. No significant gender differences were observed in awareness levels. Thus, we show the need for targeted, behavior-focused and culturally appropriate interventions to bridge the gap between knowledge and practice.

## Figures and Tables

**Table 1:** Demographic characteristics of study participants

**Demographic Variable**		**Frequency/Percent**
Gender	Male	172 (32%)
	Female	366 (68%)
	18.1 - 25 years	153 (28.4%)
Age	25.1 - 35years	133 (24.7%)
	35.1 - 45 years	145 (27%)
	45.1 - 55 years	87 (16.2%)
	55.1 - 65 years	20 (3.7)
Educational level	No school education	23 (4.3%)
	High school	197 (36.6%)
	Graduate	295 (54.8%)
	Postgraduate	23 (4.3%)
City	Al-Hufof	154 (28.6%)
	Al-Mubaraz	103 (19.1%)
	Al-Omran	277 (51.5)
	Al-Oyoon	4 (0.7%)
	Student	123 (22.9%)
Employment Status	Employed	222 (41.3%)
	Unemployed	158 (29.4%)
	Retired	35 (6.5%)

**Table 2:** Shows the activity level of the study participants

**Activity level**	**Frequency/Percent**
Sedentary (no exercise-gardening or housework etc.)	187 (34.8%)
Moderately active (3 to 5 times/week 20-30 minutes each time)	222 (41.3%)
Active (3 to 5 times/week 60 minutes each time)	73 (13.6%)
Very active (3 to 5 times/week 90 minutes each time)	30 (5.6%)
Extremely active (5 or more times /week 90 minutes plus per session)	26 (4.8%)

**Table 3:** Shows the dietary habits of study participants

**Questions regarding dietary habits of study participants**		**No**	**%**
	Vegetarian	71	13.2
Type of Diet	Non-Vegetarian	423	78.6
	Vegan	44	8.2
How many meals per day do you eat	One meal	26	4.8
	Two meal	252	46.8
	Three meals	224	41.6
	More than three meals	36	6.7
How often do you skip meals	Always	64	11.9
	Sometimes	419	77.9
	Never	55	10.2
Do you pay attention or monitor your portion sizes	Always	128	23.8
	Sometimes	309	57.4
	Never	101	18.8
What is the portion size of your meal	Very small and frequent	14	2.6
	Small	51	9.5
	Medium	429	79.7
	Large	43	8
What type of beverages do you drink	Water	507	94.2
	Milk	249	46.3
	Carbonated drinks	186	34.6
	Coffee	319	59.3
	Tea	362	67.3
	Fresh Fruit Juice	216	40.1
	Sweetened juice	93	17.3
	Energy drinks	36	6.7

**Table 4:** Anthropometric measurements of study respondents

**Variable**		**Male**	**Females**
		**Mean±SD**	**Mean±SD**
Anthropometric measurements	Height in cms	171.8 ± 7.9	157.5 ± 7.1
	Weight in kgs	84.1 ± 21.2	65.4 ± 20.0

**Table 5:** Level of awareness between males and female participants

**Type of Awareness**		**Mean**	**Standard Deviation**	**Level of Awareness**
Healthy nutritional practices	Male	2.12	±0.16	Moderate
	Female	2.13	±0.17	Moderate
Nutritional awareness	Male	2.64	±0.20	High
	Female	2.67	±0.20	High
Physical activity	Male	2.36	±0.27	High
Awareness	Female	2.36	±0.24	High

**Table 6:** The Mann-Whitney U test on gender differences of healthy nutritional awareness

**Type of Awareness**	**Mann-Whitney U**	**Sig**	**Result**
Healthy nutritional practices	28341.5	0.06NS	no differences
Nutritional	27811	0.28NS	no differences
Physical activity	30710	0.65NS	no differences

## References

[1] Mozaffarian D (2014). N Engl J Med..

[2] Naeem Z (2015). Int J Health Sci..

[3] Satija A (2016). PLoS Med..

[4] https://pubmed.ncbi.nlm.nih.gov/33069327/.

[5] Li YY (2024). Front Nutr..

[6] Hruby A, Hu FB. (2015). Pharmacoeconomics..

[7] Al-Omar H (2024). Saudi Pharm J..

[8] Althumiri NA (2021). Healthcare (Basel)..

[9] Mansour S (2024). Nutrients..

[10] Renard M (2021). Nutrients..

[11] Binobead MA (2022). Nutr Hosp..

[12] Suárez-Moreno N (2025). Nutrients..

[13] Murtaza F (2016). J Fam Community Med..

[14] Al-Neklawy AF (2025). Cureus..

[15] Frias-Toral E (2026). J Transl Med..

[16] Alshahrani JA (2026). BMC Public Health..

[17] O'Hearn M (2023). Nat Med..

[18] Stanaway JD (2022). Nat Med..

